# Understanding Organisms Using Ecological Observatory Networks

**DOI:** 10.1093/iob/obad036

**Published:** 2023-09-25

**Authors:** B Dantzer, K E Mabry, J R Bernhardt, R M Cox, C D Francis, C K Ghalambor, K L Hoke, S Jha, E Ketterson, N A Levis, K M McCain, G L Patricelli, S H Paull, N Pinter-Wollman, R J Safran, T S Schwartz, H L Throop, L Zaman, L B Martin

**Affiliations:** Department of Psychology, University of Michigan, Ann Arbor, MI 48109,USA; Department of Ecology and Evolutionary Biology, University of Michigan, Ann Arbor, MI 48109,USA; Department of Ecology and Evolutionary Biology, University of Michigan, Ann Arbor, MI 48109,USA; Department of Biology, New Mexico State University, Las Cruces, NM 88003,USA; Department of Biology, New Mexico State University, Las Cruces, NM 88003,USA; Department of Integrative Biology, University of Guelph, Guelph, ON N1G 2W1, Canada; Department of Biology, University of Virginia, Charlottesville, VA 22940,USA; Department of Biological Sciences, California Polytechnic State University, San Luis Obispo, CA 93407,USA; Department of Biological Sciences, California Polytechnic State University, San Luis Obispo, CA 93407,USA; Department of Biology, Centre for Biodiversity Dynamics (CBD), Norwegian University of Science and Technology (NTNU), N‐7491 Trondheim, Norway; Department of Biology, Centre for Biodiversity Dynamics (CBD), Norwegian University of Science and Technology (NTNU), N‐7491 Trondheim, Norway; Department of Biology, Colorado State University, Fort Collins, CO 80523, USA; Department of Biology, Colorado State University, Fort Collins, CO 80523, USA; Department of Integrative Biology, University of Texas at Austin, Austin, TX 78712,USA; Department of Biology, Indiana University, 1001 E. Third Street, Bloomington, IN 47405,USA; Department of Biology, Indiana University, 1001 E. Third Street, Bloomington, IN 47405,USA; Global Health and Infectious Disease Research Center, College of Public Health, University of South Florida, Tampa, FL 33612,USA; Department of Evolution and Ecology, University of California, Davis, CA 95616,USA; Battelle, National Ecological Observatory Network, 1685 38th Street, Boulder, CO 80301, USA; Department of Ecology and Evolutionary Biology, University of California Los Angeles, 621 Charles E. Young Drive South, Los Angeles, CA 90095, USA; Department of Ecology and Evolutionary Biology, University of Colorado, Boulder 80309,USA; Department of Biological Sciences, Auburn University, Auburn, AL 36849, USA; School of Earth and Space Exploration and School of Life Sciences, Arizona State University, Tempe, AZ 85287, USA; Department of Ecology and Evolutionary Biology, University of Michigan, Ann Arbor, MI 48109,USA; Center for the Study of Complex Systems, University of Michigan, Ann Arbor, MI 48109, USA; Global Health and Infectious Disease Research Center and Center for Genomics, College of Public Health, University of South Florida, Tampa, FL 33612,USA

## Abstract

Human activities are rapidly changing ecosystems around the world. These changes have widespread implications for the preservation of biodiversity, agricultural productivity, prevalence of zoonotic diseases, and sociopolitical conflict. To understand and improve the predictive capacity for these and other biological phenomena, some scientists are now relying on observatory networks, which are often composed of systems of sensors, teams of field researchers, and databases of abiotic and biotic measurements across multiple temporal and spatial scales. One well-known example is NEON, the US-based National Ecological Observatory Network. Although NEON and similar networks have informed studies of population, community, and ecosystem ecology for years, they have been minimally used by organismal biologists. NEON provides organismal biologists, in particular those interested in NEON's focal taxa, with an unprecedented opportunity to study phenomena such as range expansions, disease epidemics, invasive species colonization, macrophysiology, and other biological processes that fundamentally involve organismal variation. Here, we use NEON as an exemplar of the promise of observatory networks for understanding the causes and consequences of morphological, behavioral, molecular, and physiological variation among individual organisms.

## Introduction

**Fig. 1.**
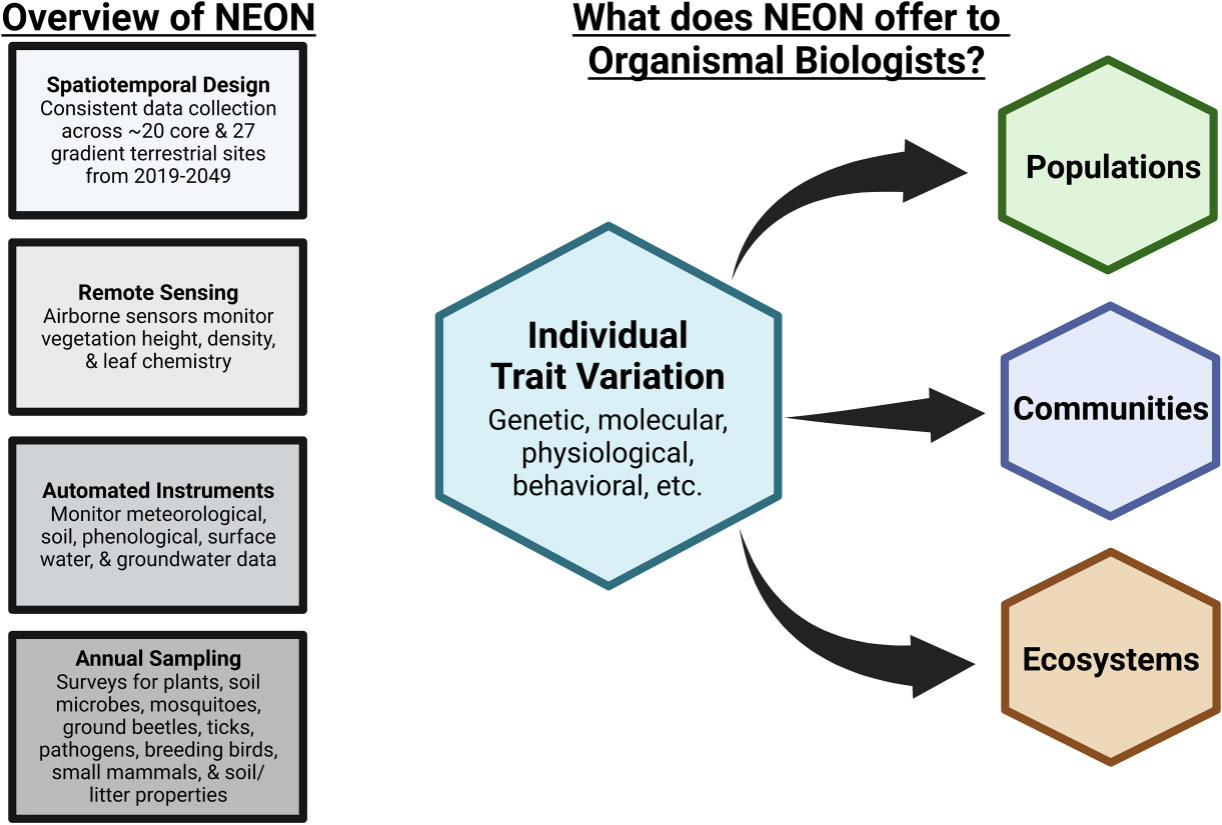
Observatory networks such as NEON provide organismal biologists with an opportunity to quantify the drivers of variation in many different organismal traits (genetic, molecular, physiological, behavioral, morphological, life history, etc.) and also how this variation may or may not scale up to influence populations, communities, or ecosystems. In the case of NEON (and many of the other observatory networks), this is possible through spatiotemporal replication, remote sensing data or those collected through automated instruments at the specific field sites, and annual sampling by observers. These data combined with archived samples collected during the annual sampling (such as at the NEON Biorepository) provide organismal biologists with many opportunities to address outstanding questions in the field centered around the causes and consequences of individual trait variation.

Many biologists seek to describe and understand how environmental change affects diversity at multiple levels of biological organization. This directive has a great sense of urgency, as anthropogenic impacts on the world are intensifying. Over the last few decades, ecological observatory networks, such as the National Ecological Observatory Network (NEON, funded by the US National Science Foundation), have been developed in part to document and provide a means to understand the effects of anthropogenic influences on ecological systems. Most work to date using data from observatory networks, such as NEON, has focused on high-level (ecological) phenomena such as metapopulation dynamics and ecosystem services (Nagy et al. 2021). However, these networks also provide a great opportunity to understand the causes and consequences of variation at lower levels of biological organization, namely genetic, molecular, physiological, and behavioral variation among individual organisms (Fig. 1). Investigating organismal variation over large spatial and temporal scales, something these networks inherently enable, holds great potential to enhance the development of theory for how and why individuals vary. Such theory and empirical insight will also have ramifications for higher-level (populations, communities, or ecosystems) processes, too, such as disease outbreaks, geographic range shifts, and community stability and productivity. Because observatory networks across the globe collect standardized data that are replicated temporally and spatially, broad-scale comparative organismal biology becomes both possible and cost-effective (Box 1).

Box 1. **What is the National Ecological Observatory Network (NEON)?** The mission of the National Ecological Observatory Network (NEON) is to collect long-term, continental-scale, open-access data and specimens with the goal of understanding the responses of terrestrial and aquatic ecosystems to environmental change (Keller et al. 2008; Schimel et al. 2011; Thorpe et al. 2016). Standardized collection protocols are designed to facilitate cross-scale analyses to address the Grand Challenges in Environmental Science (National Research Council 2001). These data are collected by teams of technicians and a range of remote/automated methods (Kitzes et al. 2021). NEON data resources include detailed data for both abiotic (e.g., climate, landscape) and biotic conditions (e.g., plant abundance, point counts of birds, small mammal mark and recapture) at 81 sites distributed across the United States. The NEON Biorepository also includes samples of soil, water, and organisms (currently > 296,000 samples from > 2,700 taxa, with > 100,000 new samples added each year) that are available for further analysis. The observatory design is well suited to investigate how factors such as invasive species, climate, and land use change influence biogeochemical, biodiversity, and infectious disease patterns.

NEON data and specimens are collected at multiple temporal and spatial scales (described in detail by Thorpe et al. 2016; Barnett et al. 2019). Temporally, data collection across all NEON sites began in 2019, although several data products and sites reach back to 2012. Collections will continue for a total of 30 years, providing an unprecedented long-term perspective. Organismal sampling tends to occur multiple times throughout the growing season at biologically relevant frequencies (often weekly/monthly intervals). The frequency of environmental data collection differs among data types, from once/year (airborne remote-sensing data) to year-round, 1-minute averages (temperature and other instrumented measurements). Spatially, NEON data are collected at a continental scale, with 47 terrestrial sites and 34 aquatic sites spread throughout the United States (Box 1, Fig. 1). Within each site, observational data are collected at multiple plots in a spatially balanced design that allows for characterization of ecological dynamics at the site scale (Fig. 1), with the number of plots varying depending on the organism of interest (e.g., 6 plots for ticks, 10 plots for mosquitoes, 3–8 plots for small mammals). Many variables of interest about individual animals (e.g., body size, breeding phenology, various tissue samples) and plants (e.g., diameter at breast height, tree crown height and area, leaf size and chemistry) are collected, with repeated samples when individual identification is possible (e.g., for small mammals, trees, and other tagged plants). Numerous abiotic variables (e.g., temperature, precipitation, wind speed, soil heat flux, and carbon/water flux) are also measured at different heights along a tower located near the center of the site. Data collected by NEON undergo a quality assurance procedure (McCord et al. 2021), are freely accessible on their website (https://www.neonscience.org), and open-source workflows to analyze NEON data are available (Li et al. 2022). Independent researchers also have the opportunity to collaborate with NEON through the *Assignable Asset Program* (https://www.neonscience.org/resources/research-support), or provide additional funding that allows for supplemental data or sample collection and/or processing and/or the use of additional instrumentation (SanClements et al. 2020; BioScience).

**Box 1, Fig. 1.**
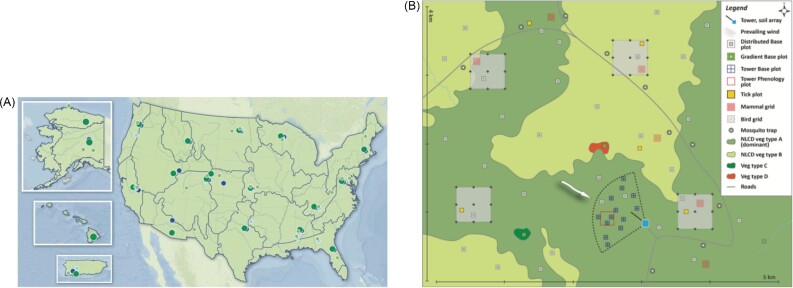
Spatial scales of NEON sampling. (**A**) The distribution of NEON sites across ecoregion boundaries in the United States (insets show Alaska, Hawaii, and Puerto Rico). Terrestrial sites are in green, while aquatic sites are in blue; larger, darker circles show NEON Core Sites (which are natural and undisturbed), while smaller, lighter circles show NEON Gradient Sites (which are impacted by human activities). Each site consists of an array of embedded plots at which sampling or automated data collection occur. See interactive map here: https://www.neonscience.org/field-sites/explore-field-sites. (**B**) An expanded view of a typical NEON Core terrestrial site containing multiple types of data collection.

Modern organismal biologists use diverse, integrative approaches to understand how variation in the phenotypes of individuals manifests and scales up to affect population, community, and ecosystem processes (Fig. 1). For example, a recent interest in animal personality traits, or consistent individual differences in behavior, has revealed that individual variation in foraging activities can influence community-level phenomena such as succession by changing which kinds of and where seeds are dispersed across a landscape (Zwolak and Sih 2020; Brehm and Mortelitti 2022). Likewise, physiological responses to ecological stressors can scale up to alter community or ecosystem-level processes. One example involves prey physiological responses to predation risk, which can alter the nutrient composition of prey excreta (Hawlena and Schmitz 2010; Hawlena et al. 2012). Another involves the effects

of natural or anthropogenic stressors on host responses to infection; the spread and/or persistence of zoonotic disease risk can change depending on the magnitude and duration of stressors and resultant effects on host attractiveness to vectors and the ability of individual hosts to transmit viruses and other pathogens (Kernbach et al. 2019; Martin et al. 2019).

Arguably, organismal biology is particularly well positioned to identify where individual trait variation comes from and why it matters (Wake 2008; Stillman et al. 2011; Kültz et al. 2013; Martin et al. 2014). While incorporating such individual-level variation would appear necessary for many aims, often studies treat individuals as functionally identical. Indeed, most models forecasting global climate change impacts on biodiversity do not consider individual-level trait variation (Huey et al. 2012; Somero 2012; Nemeth et al. 2013; Urban et al. 2016). This absence of attention is important to address given the above results as well as some theoretical studies showing that the probability of population extinction can be influenced by individual-level characteristics (Botero et al. 2015). Because organismal biologists naturally focus on the numerous and diverse mechanisms by which organisms cope with change, which underpins individual-level variation (Somero 2010; Urban et al. 2016), organismal approaches will generate the requisite data needed to parameterize effective models. Such models should reveal more basic and management-directed insight than is possible using approaches that ignore individual-level variation.

A major challenge in organismal biology, however, is that practitioners are often logistically and financially constrained and lack the appropriate resources and infrastructure required for larger-scale spatiotemporal replication (Clutton-Brock and Sheldon 2010; Reinke et al. 2019; Sheldon et al. 2022). While for some questions in organismal biology, spatiotemporal replication will not be necessary, for many others, comparative work with individuals spread over broad ranges will not only be interesting, but also imperative. Observatory networks enable organismal research that covers both broad spatial and temporal scales. They will also provide an unprecedented opportunity to do novel, integrative biology in the historic natural settings in which populations evolved and the intensely human-modified contexts that dominate much of the landscape today. We believe the time has come for organismal biology to take full advantage of observatory networks, a view espoused by others regarding the outcomes of biological invasions (Gill et al. 2021) and the identification of “tipping points” in ecosystems (Muthukrishnan et al. 2022). Below we discuss the potential value of observatory networks to organismal biology. Although we focus on NEON, our views also apply to other observatory networks, and space constraints prevent us from exploring all of the nuanced differences (and similarities) among observatory networks.

## Using NEON to understand the individual organism in its environment

**Fig. 2.**
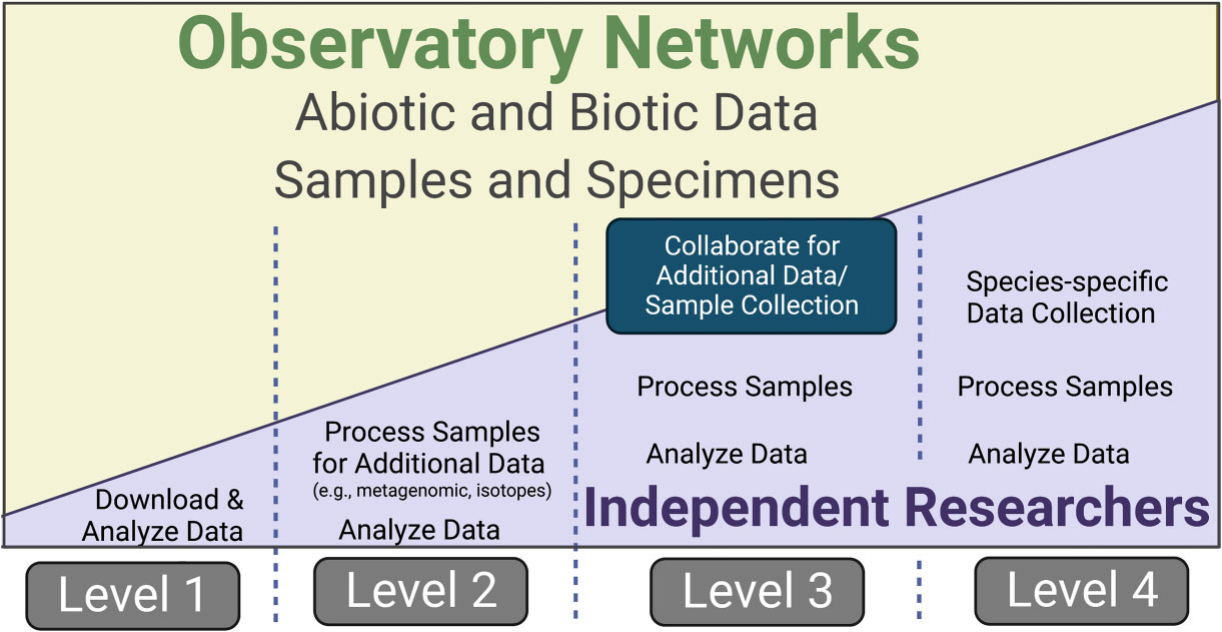
The relative contributions of resources by NEON or other observatory networks (yellow shaded area) and independent researchers (purple shaded area) to specific types of projects vary. At Level 1, researchers largely use existing data collected by NEON to address outstanding questions in their field. At Level 2, researchers may use samples housed at the NEON Biorepository to address their specific research questions. At Levels 3 and 4, researchers may need to either collaborate with NEON or work independently at or near NEON sites to collect additional data. For instance, independent researchers could collect additional data at NEON sites (Level 3) or focus on a species of interest by setting up their own study site adjacent to NEON sites (Level 4). Although these collaborations hold much potential, it will require NEON to work with independent researchers to collect additional data to address their specific research questions (e.g., through the Assignable Assets Program offered by NEON).

Like other observatory networks, NEON collects abiotic and biotic data at different spatial and temporal scales and curates biological samples from individuals of several widespread taxa along with a variety of environmental samples (Box 1, Fig. 1; Table S1). Data currently collected by NEON provide ample opportunities for organismal biologists, but more collaborations among researchers are needed, possible, and promising (Fig. 2). The first and simplest research path for organismal biologists entails analyses of existing data freely available from the NEON data portal (https://data.neonscience.org; Level 1 in Fig. 2). A second option requires that individual researchers (who are independent from NEON) generate new data from biological samples stored at the NEON Biorepository located at Arizona State University (https://biorepo.neonscience.org/portal/index.php; Level 2 in Fig. 2). Perhaps, the most involved and yet tractable projects would entail individual researchers (again, independent from NEON) collecting additional data at NEON sites (Level 3 in  Fig. 2) or focusing on a species of interest by setting up their own study sites adjacent to NEON sites (Level 4 in Fig. 2), both of which can be facilitated via the NEON Assignable Assets program. The latter (especially Level 3 in Fig. 2) would provide access to the rich environmental data NEON collects while enabling individual researchers the opportunity to carry out their own observational or experimental work (in the case of Level 4 in Fig. 2) on a study species that may not be focal to NEON's collections. Beyond collecting their own data, researchers could use existing colocated datasets (Table S2; Nagy et al. 2021), which could further expand the scope of organismal biology possible via observatory networks.

To make the case more explicit for the use of NEON by organismal biologists, we offer some examples. For instance, using currently available data, one could analyze biogeographic patterns in existing morphometric or biometric data (e.g., growth and phenology of many species; Level 1 in Fig. 2). For the plant, invertebrate, and vertebrate species that NEON has prioritized (i.e., species that are widely distributed across sites and abundant within sites), more involved studies are possible through processing previously collected samples or specimens (Level 2 in Fig. 2). For NEON focal species that are broadly distributed, or to enable large-scale comparisons of similar taxa, one might compare the transcriptomic or proteomic response to a salient but spatiotemporally broad factor (e.g., photoperiod, climate, various forms of pollution). Resultant data could reveal the extent to which the expression of genes or phenotypes varies with the environment, the extent to which variable environments promote the evolution of phenotypic plasticity, or the extent of trait covariation within individuals. These studies could also use NEON data to identify study sites at which individual researchers could perform additional sampling (i.e., study sites that experience the highest and lowest levels of variability in an abiotic variable of interest) for their specific research questions.

Another potential research opportunity could leverage data resulting from NEON's standardized fish sampling (Table S1), paired with their river, stream, or lake sampling programs (e.g., aquatic plant and macroalgal point counts, benthic macroinvertebrate community metrics, and riparian structure and vegetation measures) to study the drivers of intraspecific variation in morphological traits (Level 1 in Fig. 2). Fin clip samples are collected from captured fish at one time point (individuals are typically not captured more than once) and stored in the NEON Biorepository. These samples offer additional opportunities to evaluate relationships among morphology, body condition, genetic variation, and resource use (e.g., from stable isotope analysis) as a function of environment among individuals (Level 2 in Fig. 2) and populations. To date, comparable work on the drivers of individual phenotypic variation in fish have tended to focus on one or a few specific environmental axes (e.g., Colosimo et al. 2005; Lofeu et al. 2021; Ronco et al. 2021). NEON, in contrast, offers the opportunity to quantify the contributions of multiple biotic (e.g., competitors, available food, population size and structure) and abiotic (e.g., temperature, pH, and turbidity) factors (Box 1), and their interactions, to phenotypic variation across space.

Key questions in infectious disease biology would also benefit from NEON's unique spatial and temporal replication. One emerging topic that is gaining importance involves how environmental heterogeneity gives rise to individual heterogeneity in hosts, pathogens, or vector traits, which then alters disease risk for the community over space and time (Cook et al. 2016; Springer et al. 2016; Klarenberg and Wisely 2019; Paull et al. 2022). Convention, especially in epidemiological modeling efforts, has been to consider individuals as homogeneous in their susceptibility to acquire and transmit infectious organisms. More recently, this practical shortcut has been revised due to the recurring observation of a Pareto-type distribution of infectiousness for most individuals. In other words, 20% of individuals tend to cause 80% of infections (Hawley and Altizer 2011; Lively et al. 2014; Martin et al. 2019). Focusing just on small mammals sampled within NEON (e.g., Read et al. 2018; Guralnick et al. 2020; McLean and Guralnick 2021), one could integrate individual host phenotype, community diversity, and infection type, and burden data to probe how organismal variation affects risk of tick-borne infections (Levels 2–4 in Fig. 2; Klarenberg and Wisely 2019; Paull et al. 2022).

Some researchers are already using NEON in a manner amenable to organismal biology. For example, global climate change is driving phenological shifts (e.g., onset of breeding in seasonal breeders, length of breeding, or growing season) in many species. In a few plant and animal species spread across North America, NEON has documented temporal changes in the start and end of growing seasons in 17 different ecoclimatic domains (Liang et al. 2021). Now, organismal biologists could get involved to identify the relative effects of different abiotic features (temperature, precipitation, and photoperiod) on breeding phenology in small mammals and the various mechanisms by which they arise (McLean and Guralnick 2021). Indeed, an ongoing problem is understanding how phenological changes arise mechanistically in most taxa (e.g., Cleland et al. 2007; Renner and Zohner 2018; Li et al. 2019; Visser and Gienapp 2019; Satake et al. 2022). NEON, especially the Biorepository, could help facilitate research in this important area. For instance, existing efforts to document changes in phenology (Liang et al. 2021; McLean and Guralnick 2021) could be merged with molecular and physiological efforts and even coupled with other phenology network data (e.g., USA National Phenology Network: https://www.usanpn.org) or colocated datasets (Table S2). This nationally distributed, integrative work could help us broadly discern whether and how individuals integrate environmental cues to regulate their phenology. Plant research should fare particularly well in this frame because documenting changes in plant phenology may be easier than in many animal species because the relative immobility of plants provides the possibility of repeated observations of the same individual plants or populations across temporal scales. Plant organismal biology is particularly facilitated by NEON's Phenocam data, which entails time-lapsed digital photographs of plants. Phenocam data from NEON (Seyednasrollah et al. 2019, 2020) paired with individual-based observations or measurements of plant or soil chemistry could show whether vegetative and reproductive biomass investment of individuals within populations respond differently to local climate. The pairing of these efforts with plant and pollinator data (e.g., Donnelly and Yu 2021) could further elucidate the causes of variation in plant reproduction (Levels 3 and 4 in Fig. 2), just as genetic and/or epigenetic sequencing efforts could advance our understanding of the relative roles of molecular regulatory mechanisms in individual variation in different plant traits (Roux et al. 2006; Alonso-Blanco et al. 2009; Level 2 in Fig. 2).

NEON's infrastructure could potentially facilitate the study of the organismal biology of nonfocal taxa, too. For example, the addition of automated recording units (ARUs) for acoustic surveillance by researchers at NEON sites (Levels 3 and 4 in Fig. 2) could lead to novel insight into the biology of birds, frogs, insects, and probably other species (Buxton et al. 2018; Yip et al. 2021). Automated recording units are currently not deployed at any NEON site, but if deployed and used by individual researchers, they would capture the arrival of individual animals at breeding sites, the timing of their breeding behavior (e.g., Buxton et al. 2016; Oliver et al. 2018), and/or changes in daily activity patterns (Bradfer-Lawrence et al. 2019). These acoustic data could then be placed into an ecological context using NEON environmental data or further studied in relation to physiological, molecular, or behavioral traits of focal individuals or species. Similarly, individual researchers could use their own independent funding to collaborate with NEON to deploy wildlife camera traps across NEON sites to investigate the behavior of mesocarnivores, ungulates, and other terrestrial vertebrates at a continental scale. Such an effort would in some ways be similar to existing projects such as Snapshot USA (Cove et al. 2021; Kays et al. 2022), but an explicit difference would be the availability of tremendous amounts of associated abiotic and biotic data collected by NEON.

## Current challenges of observatory networks for organismal biology

The large-scale nature of the NEON project inevitably subjects it to criticism resulting from the diversity of perspectives and methodologies inherent to various subdisciplines of biology (Lindenmayer and Likens 2009; Lindenmayer et al. 2018; Knapp and Collins 2019; Sagoff 2019). Revisiting these concerns is not the goal here. Instead, we want to highlight some specific gaps in current NEON practices from the perspective of organismal biology and provide suggested researcher-driven remedies to facilitate research in these study areas, although we are sure our list is not exhaustive. We note that some of these challenges are specific to NEON, but many are applicable to other observatory networks. Ideally, this discussion of these challenges helps to drive improvements in the design of future observatory networks.

First, there is presently an almost complete dearth of behavioral data for any NEON focal species. One conspicuous and actionable exception is the live-trapping data for individually identified small mammals, which enable study of individual home range size and other aspects of space use. The relative absence of behavioral data is not surprising considering the high costs of acquiring and analyzing such data. However, this absence is concerning because behavior is a key component of how animals respond to and cope with environmental change (Bartholomew 1964; Snell-Rood 2013; Sih 2013). NEON data are not collected only through remote sensing, but through labor-intensive field surveys by scores of field technicians (Box 1; Table S1). Researchers have the opportunity to bring additional funds that could capitalize on NEON's existing infrastructure and also support collection of behavioral data that is outside the current scope of NEON's mandate.

Second, many critical taxa are excluded from the current sampling design of NEON (Kitzes et al. 2021). This issue could partially be resolved by deploying camera traps, ARUs, hair snares, or eDNA sampling from pitfall traps that NEON uses to sample invertebrates (Weiser et al. 2022). Based on related work, camera trapping should be especially useful to characterize the abundance, distribution, and some behaviors of large terrestrial vertebrates (Rovero and Marshall 2009; Rovero et al. 2013; Rowcliffe et al. 2014; Steenweg et al. 2017; Smith et al. 2020). Other taxa might simply remain too costly or challenging to study and require independent researchers to conduct their own studies near NEON sites (Level 4 in Fig. 2).

Third, quantification of microenvironmental variation at NEON sites is largely nonexistent, although such fine-scale data are integral to understanding many organismal phenomena (Kearney and Porter 2009). Identifying the scale of microenvironmental variation relative to larger scale environmental variation at NEON sites may require additional sampling by independent researchers, such as deploying monitoring devices at the scale of the study organism of interest. On the other hand, a recent study that deployed multiple temperature sensors across a study site highlighted that remote sensing data (airborne LiDAR) collected by NEON can be used to estimate within-site or microenvironmental variability in maximum and minimum temperatures (Davis et al. 2019). This result provides some optimism, but will require ground truthing from organismal biologists for their specific study species.

Fourth, the multidecade timescale of NEON presents an exceptional opportunity to link rich, hyperdimensional descriptions of ecological change with simultaneous characterizations of evolution as it happens. Combining ecological and evolutionary dimensions will be particularly critical for understanding how populations respond to climate change. Although inferences about evolution in response to climate change can be drawn from phenotypic data, additional genetic (and epigenetic) data can greatly strengthen evolutionary inferences and distinguish genetic adaptation from phenotypic plasticity (Gienapp et al. 2008; Merila 2012; Merila and Hendry 2014; McGuigan et al. 2021). However, the genetic data currently available from NEON include sequences for specific marker genes (e.g., CO1 for small mammals, fish, beetles, mosquitos, zooplankton, and aquatic macroinvertebrates; 16S rRNA sequences for soil and aquatic microbes), as well as metabarcoding datasets that are designed to describe species composition, not to characterize genetic variation or track evolutionary change within populations. Instead, what is needed are studies that track candidate phenotypic traits and loci thought to be under selection across NEON sites and how these traits and genes are changing over time. Thus, there is an opportunity for organismal biologists to establish their own projects (Levels 2–4 in Fig. 2) quantifying how genetic and phenotypic changes are occurring within the rich ecological context provided by NEON.

Finally, NEON may hold even greater promise if independent researchers aspire to integrate the local and traditional knowledge of Indigenous communities where NEON sites are located. Guidelines described elsewhere could foster such work (e.g., Wong et al. 2020), and collaborations with hunters, fishers, trappers, and those who have local knowledge about the number and type of animals harvested could give both depth and scope to the projects possible at NEON sites. One mutually beneficial type of project would document the causes of changes in large terrestrial vertebrates (i.e., natural or anthropogenic activities), performed in a mutualistic way, conscientious and respectful to the beliefs and attitudes of those who choose to participate.

## Conclusions

Observatory networks across the globe represent unprecedented, collaborative opportunities for integrative organismal biology. Clearly, substantial efforts are required to understand how organismal variation arises and how such variation affects populations, communities, and ecosystems in the face of rapid environmental change. NEON, along with other observatory networks, has the potential to augment organismal biology in important ways. Although we have focused on NEON, our points apply to many other observatory networks. We also hope our perspective is useful in the development of any new observatory networks. Ultimately, observatory networks provide a figurative and literal nexus of collaborative opportunity between organismal and other biologists, but also the resource managers, network administrators, and members of the public. Such assets should be leveraged to their fullest.

## Supplementary Material

obad036_Supplemental_File
