# Supplementary material for: Understanding Organisms Using Ecological Observatory Networks
Source: Integr Org Biol. 2023 Sep 25;5(1):obad036. doi: 10.1093/iob/obad036 (PMC10586040; doi:10.1093/iob/obad036)
Supplement: obad036_Supplemental_File [file obad036_supplemental_file.docx]

***Understanding organisms using ecological observatory networks***

Ben Dantzer, Karen E. Mabry, Joey R. Bernhardt, Robert M. Cox, Clinton D. Francis, Cameron K. Ghalambor, Kim L. Hoke, Shalene Jha, Ellen Ketterson, Nicholas A. Levis, Kailey M. McCain, Gail L. Patricelli, Sara H. Paull, Noa Pinter-Wollman, Rebecca J. Safran, Tonia S. Schwartz, Heather L. Throop, Luis Zaman, Lynn B. Martin

**Table S1.** Representative NEON data and collections (datasets available for immediate analysis and samples and specimens available for data extraction) with examples of how they may be used in studies of organismal biology. Here we focus specifically on the types of data that would be useful for organismal biologists (see also Box 1 in Nagy et al. 2021). This list is not exhaustive and additional information about NEON sampling and data is available elsewhere (SanClements et al., 2020).

| **Data set** | **Potential questions to be addressed** | **Example biorepository samples associated with the data set** | **Potential questions to be addressed with additional sample processing** |
| --- | --- | --- | --- |
| Small mammal live-trapping and mark-recapture | Comparisons of home-range area, local activity patterns in time and space, and trappability as a measure of exploratory/bold behavior across gradients of ecotype and disturbance. | Small mammal fecal samples | Assay hormones to assess reproductive state and health among individuals and locations; relate to activity patterns, morphometrics and tick load. Use metagenomics to identify parasites and diet and how these change with invasive species presence. |
| Small mammal morphometrics | Inference about metabolic rate and condition from body mass and hind foot length; field assessment of reproductive condition to describe phenology of breeding. | Small mammal hair and whisker samples | Use stable isotopes to estimate trophic level and niche specialization, quantify steroid hormones. |
| Small mammal tick infestation and tick-borne pathogens | Examine relationships between environmental gradients and tick-borne pathogen prevalence. | Small mammal ear and blood samples as well as whole (opportunistic) vouchers (skin, skeleton, heart, kidney, liver, lung, spleen, muscle, ecto/endo parasites). Archived tick specimens collected via drag sampling at the site. | Compare variation in gene expression, genome, methylation, and pollutants such as pesticides and heavy metals samples among sites. Use genomic analysis of mammal and tick samples to study host-parasite coevolution. |
| Fish length and mass | Evaluate the impacts of biotic and abiotic stream properties on morphological variation over space and time. | Formalin-preserved whole fish vouchers and fin clips | Focal genetic analyses on populations; do local diet, pollutants and predation pressure affect population genetic structure and hence viability and evolvability? |
| Mosquitoes sampled with CO_2_ traps and associated pathogens | Compare how mosquito life history influences phenological shifts within and among species. Examine mosquito-transmitted pathogen presence relative to avian host community traits across space and time. | Preserved mosquitoes (pinned specimens and bulk archived specimens grouped by species, site, collection bout and sex) | Examine distributions, population genetic, and phenotypic variation relative to microclimatic variation and longer-term extreme climate events, (e.g., droughts). |
| Ticks sampled using drag cloths and associated pathogens | Examine environment and host community composition influences on tick diversity, abundance and pathogen prevalence. | Preserved individual ticks | Examine effects of local climate on tick reproductive traits and propensity to act as infection reservoirs . |
| Ground beetle pitfall trapping | Examine beetle diversity and abundance over time and among habitats, relative to abundance and activity of small mammal and bird predators, or anthropogenic impacts on surrounding area. | Preserved pinned beetles. Use eDNA obtained from ethanol containing pitfall specimens to measure species richness. | Examine distributions, population genetic, and phenotypic variation relative to microclimates and longer-term patterns, such as droughts. |
| Zooplankton and benthic macroinvertebrate counts | Examine bottom-up effects of stream macroinvertebrate community on terrestrial mosquito and bird abundance. | Preserved zooplankton and macroinvertebrates |  |
| Growth and morphology of herbaceous plants | Phenotypic plasticity in plant form and growth among sites differing in soils, climate, and communities of plants and pollinators. | Plant voucher specimens | Studies of genomic variation within and among populations in genes associated with heat and drought tolerance. |
|  |  | Water or soil samples | Use metagenomic barcoding for community analyses, contaminants/pesticides as environmental stressors, and agents of selection. Use eDNA analyses to detect other native and invasive species or identify the predator community. |

**Table S2.** A diversity of other observatory networks and community science endeavors (iNaturalist, eBird) exist whose data can be integrated with those provided by NEON. Many of these were described elsewhere (Nagy et al., 2021), so we indicate which additional ones we added to this list using the superscript #. Some of these co-located datasets are managed by research networks, which can be an amalgam of governmental scientists and staff, non-profit organizations, and community scientists. Other data are collected by community science initiatives or governmental organizations (National Aeronautics and Space Administration, NASA) in the USA. We note that some of these data are collected as a part of a community science program (iNaturalist) that may have biased sampling. This list is non-exhaustive and we have focused on entities that use similar data collection mechanisms as NEON.

| **Supplemental Co-located data** | **Organization** | **Research Foci** |
| --- | --- | --- |
| LTER* | Research Network | Population, community and ecosystem-level processes; some organism-level data depending on site |
| PhenoCam* | Research Network | Phenological shifts over space and time |
| USA National Phenology Network | Research Network | Phenological shifts over space and time |
| Nutrient Network (NutNet)# | Research Network | Environment-productivity-diversity relationship |
| DragNet# | Research Network | Herbaceous plant community dynamics |
| Landsat/MODIS | NASA | Global-scale environmental change |
| Global Ecosystem Dynamics Investigation (GEDI) | NASA | Forest canopy structure that could influence how organisms interact |
| iNaturalist# | Community Science | Location data for plants and animals |
| eBird# | Community Science | Bird location data |
| Moonlight data# | R package Moonlit^A^ | Among-site variability in moonlight |
| Light pollution# | NASA/NOAA VIIRs^B^ | Among-site variability in light pollution |

* - data already exists at a subset of NEON sites

# - not mentioned in Nagy et al. (2021)

^A^R Computing Software package available at <https://github.com/msmielak/moonlit>

^B^Visible Infrared Imaging Radiometer Suite (VIIRS) instrument on the joint NASA/NOAA Suomi National Polar-orbiting Partnership. See Falchi et al. (2016)
